# Unlikely Storyteller: Leveraging Narrative-Based Communication in LLM-Generated Medical Advice

**DOI:** 10.3390/healthcare14081015

**Published:** 2026-04-13

**Authors:** Fan Wang, Ningshen Wang, Weiming Xu, Peng Zhang

**Affiliations:** 1 Foreign Languages College, Shanghai Normal University, 100 Guilin Road, Xuhui District, Shanghai 200233, China; candywang@shnu.edu.cn; 2 Department of Mechanical Engineering, City University of Hong Kong, 83 Tat Chee Avenue, Kowloon Tang, Kowloon, Hongkong 999077, China; ningswang4-c@my.cityu.edu.hk (N.W.); weimingxu3-c@my.cityu.edu.hk (W.X.)

**Keywords:** large language models (LLMs), narrative medicine, patient-centered communication, prompt engineering, algorithm aversion

## Abstract

Background/Objectives: Time-constrained consultations in high-volume settings can crowd out patient-centered communication, while AI-generated advice may face algorithm aversion when it lacks a humanistic dimension. This study examined whether a brief narrative-based prompt could improve coded patient-facing communication features in an LLM relative to both clinicians and an unprompted model on authentic patient queries. Methods: We conducted a three-condition comparative evaluation using a stratified sample of 1000 de-identified MedDialog-CN consultations (2016–2020). For each consultation, the same patient query was used to generate (i) a zero-shot GPT-o3-mini response and (ii) a narrative-prompted GPT-o3-mini response; the original physician reply served as the human baseline. Responses were annotated with a pre-specified schema operationalizing four communication dimensions—Storytelling, Empathy, Personalization, and Clarity—with expert adjudication. Frequency-based indicators were summarized as mean events per consultation, and binary indicators as proportions; secondary checks captured unwarranted certainty and risk-relevant language. Results: Narrative prompting shifted coded patient-facing communication from sparse and selectively deployed (clinicians and zero-shot AI) to more routine and standardized. Across the reported communication measures, the prompted model showed the most favorable overall pattern, with higher narrative-device use, empathic support, contextual tailoring, and terminology explanation, alongside more frequent consideration of patient preferences and markedly higher rates of emotion–symptom linkage and the presence of a patient-centered narrative framework. Conclusions: Narrative prompting may offer a lightweight and potentially scalable strategy for improving patient-facing communication in Chinese asynchronous, text-based online consultations. An important next step is calibration: humanistic cues should be delivered selectively and safely so that responses remain credible, locally feasible, and cognitively manageable.

## 1. Introduction

Time pressure can erode the relational and explanatory features that underpin patient-centered care. In China, a typical outpatient neurology visit lasts barely three minutes [1], and consultations on online medical platforms such as Haodf.com are often even shorter. Such brevity leaves little room for the empathy, personal framing and jargon-free explanation that anchor patient-centered care [2,3], although decades of evidence show that integrating storytelling, empathy, personalization, and clarity improves adherence and clinical outcomes [4,5]. Yet workload and burnout steadily push both offline and online encounters toward terse, data-heavy prose [6,7].

Large language models (LLMs) are increasingly discussed as tools that may help relieve some of this pressure. ChatGPT-o3-mini now performs strongly on a range of diagnostic reasoning benchmarks [8], and recent work points to possible gains in efficiency, accessibility, and clinical decision support [9,10]. From interpreting diagnostic images to generating personalized treatment plans, AI systems are rapidly becoming integral to modern medical practice [8,11], and have entered the realm of real-time medical advice generation [12]. Recent syntheses also emphasize that successful integration of AI into healthcare depends not only on model capability, but on implementation constraints—particularly personalization demands, users’ digital literacy, and privacy/ethical governance—which can shape real-world uptake and user experience [13]. These technologies promise to alleviate healthcare bottlenecks, particularly in areas where access to medical professionals is limited [14]. By swiftly analyzing patient data and providing accurate, data-driven recommendations, LLMs offer viable solutions to growing pressures on healthcare systems [11,15].

However, the moment advice is revealed as algorithmic, patient acceptance drops—a phenomenon known as algorithm aversion [16,17]. Patients exhibit significant resistance to AI-generated medical advice, especially when its role is explicitly disclosed [18]. This resistance is also shaped by users’ mental models of AI and the narratives through which AI is introduced. In an experiment holding the conversational agent constant, Pataranutaporn et al. [19] showed that priming participants to view the same AI as having “caring motives” increased perceived trustworthiness, empathy, and effectiveness relative to neutral or manipulative framings, highlighting that perceived humanistic intent can systematically alter how AI advice is received. Hesitancy peaks in high-volume settings where relational cues are already scarce [16]. In such environments, AI’s perceived lack of human touch can heighten skepticism, preventing its widespread use—even when it offers accurate diagnoses [20]. These findings suggest that technical accuracy alone may not be sufficient for uptake; patient acceptance also depends on whether advice is communicated in a way that appears relationally attentive and understandable [21]. This emphasis on the relational “how” is consistent with recent syntheses on AI and the doctor–patient relationship, which highlight trust, empathy/compassion, effective communication, ethics, and transparency as central values for mitigating risks as AI is integrated into clinical practice [22]. In other words, the adoption problem is not only technical; it is also relational and communicative.

Narrative-based communication, rooted in the principles of narrative medicine, offers one possible response. Metaphor, explicit empathy, context-tailored framing, and jargon translation turn biomedical facts into stories that patients can inhabit [23,24]. If those same cues could be induced in an LLM with a succinct prompt, the machine might overcome aversion. That hypothesis, however, has not been tested against both frontline clinicians and an unprompted model on authentic patient queries. Specifically, whether a concise prompt can elicit these same cues from an LLM—and thereby surpass an unprompted model and practicing clinicians on authentic patient queries—has not yet been empirically examined.

Based on this rationale, we advanced two hypotheses.

**H1:**  *Relative to the unprompted model, the narrative-prompted model would demonstrate stronger patient-facing communication across the four focal dimensions of storytelling, empathy, personalization, and clarity.*

**H2:**  *Relative to the original physician replies, the narrative-prompted model would also show stronger performance across these same dimensions. We tested these hypotheses using a three-condition comparative design in which the same patient query served as the shared input across both AI conditions.*

To test these hypotheses, we drew 1000 real doctor-patient conversations from MedDialog-CN [25], the largest corpus of Chinese online medical consultations, and resubmitted each patient query under two AI conditions: GPT-o3-mini in zero-shot mode (hereafter, “unprompted model”) and GPT-o3-mini preceded by a short paragraph of narrative prompt that cues storytelling, empathy, personalization, and clarity (hereafter, “prompted model”). We then compared both sets of AI replies with the original physicians’ answers, scoring each response on four validated communication dimensions. Our analyses showed that the prompted model produced more verbal empathic support, more terminology explanation, and the most favorable overall pattern across the reported communication dimensions. In just a brief prompt, a high-performing algorithm could be steered toward more narratively and relationally enriched patient-facing communication, pointing to a potentially useful design strategy for text-based telemedicine environments similar to the present dataset.

## 2. Methodology

**Figure 1:**
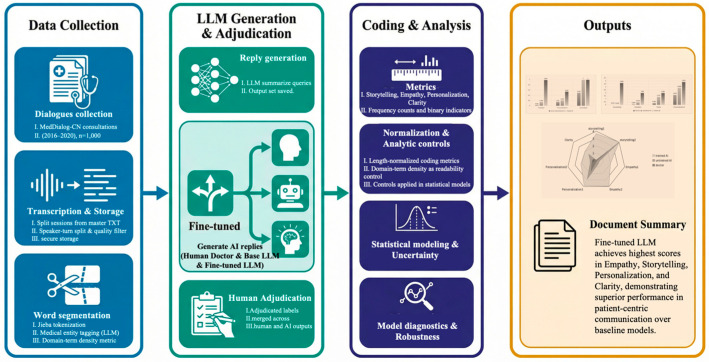
Study design and analysis pipeline.

As shown in Figure 1, we conducted a three-condition comparative evaluation using a stratified, de-identified sample of MedDialog-CN online consultations from 2016–2020 (*n* = 1000). For each consultation, the patient query served as a shared input for two LLM responses generated under matched conditions: (i) a zero-shot response (unprompted model) and (ii) a narrative-prompted response. The original physician reply served as the human baseline, so each consultation yielded a triad of responses for analysis. All responses were annotated with a pre-specified coding schema covering four communication dimensions (storytelling, empathy, personalization, and clarity) and then consolidated through expert adjudication into a unified analytic dataset. Primary outcomes were the four dimension-specific scores and their composite. The sections below detail data preparation, LLM response generation, annotation and adjudication, and the quantitative analyses used to compare the three conditions.

### 2.1. Data Collection and Preparation

We used MedDialog-CN, a publicly available corpus of de-identified Chinese online medical consultations collected from haodf.com and released for research use [25]. From this source corpus, we constructed a stratified analytic sample to ensure temporal representativeness across recent years. Specifically, we restricted the sampling frame to consultations dated 2016–2020 and selected 200 consultations per year via stratified random sampling, yielding a balanced dataset of *n* = 1000 consultations.

Inclusion and exclusion criteria were applied prior to sampling to ensure minimal interactional substance. We retained only consultations containing at least two back-and-forth rounds between patient and physician. Here, a round was operationalized as a paired exchange consisting of a patient message followed by a physician reply; thus, eligible consultations required ≥2 patient turns and ≥2 physician turns (i.e., ≥4 total turns) after session segmentation and speaker identification. Consultations were excluded if they lacked a physician reply, contained only a single exchange, or could not be reliably segmented into speaker turns due to formatting irregularities in the raw logs.

Finally, we note several metadata constraints that are typical of large-scale platform corpora and are relevant for interpretation. Clinical specialty information is not consistently available at the consultation level in the released dataset; accordingly, analyses were conducted without specialty stratification. Physician identity is also not treated as a stable, linkable identifier across consultations in the analytic dataset, so each consultation was analyzed as an independent unit without assuming repeated measures by the same clinician. Likewise, although consultations are time-stamped, the dataset does not allow us to reconstruct the interval between patient posting and physician response reliably; our comparisons therefore focus on response content and communication features rather than turnaround time.

In addition, because MedDialog-CN is a platform-based, question-led consultation corpus, reliable case-level disease categorization was not consistently possible in the present sample. Many consultations are prediagnostic and do not contain a definitive disease label, while consultation-level specialty metadata are not sufficiently stable to support robust disease- or specialty-stratified analysis. We therefore treated the corpus as clinically heterogeneous and did not model disease category as an analytic factor. Instead, the principal AI comparison was structured within consultation, with the same patient query serving as the shared input across both AI conditions, while domain-term density was included as a proxy for variation in medical complexity.

### 2.2. LLM-Assisted Generation and Adjudication

**Figure 2:**
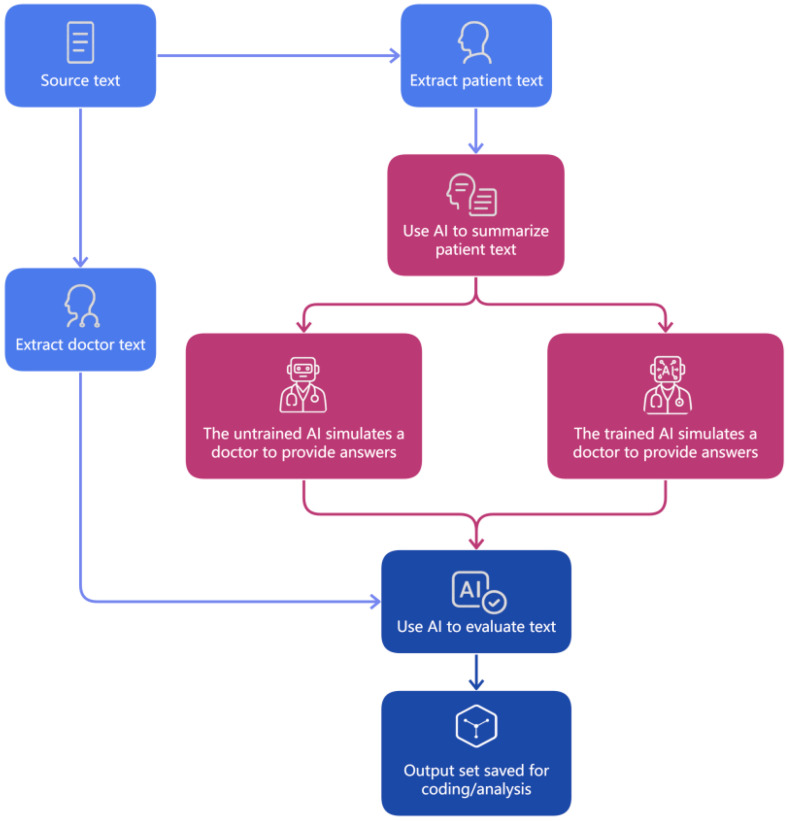
LLM generation and evaluation workflow.

Figure 2 illustrates the overall workflow of this process. For each patient query, an LLM-generated summary was first produced and used to generate candidate AI replies under two conditions: one without any prompting (unprompted model) and the other with a detailed, task-specific narrative prompt (prompted model). All LLM responses were generated using GPT-o3-mini as a fixed baseline model so that observed differences could be interpreted primarily in relation to prompt design rather than cross-model variation. A fixed temperature setting (temperature = 1) was applied uniformly across both AI conditions to maintain a common generation environment throughout the comparison. AI-generated replies, together with the original doctor responses, were compiled into a unified corpus for subsequent coding.

An AI annotator, operating under a predefined coding schema, assigned provisional labels to all responses across four narrative dimensions: storytelling, empathy, personalization, and clarity. The coding rubric was designed in advance by domain experts, who also reviewed the provisional annotations, resolved inconsistencies, and applied corrections where necessary.

More specifically, the annotation workflow proceeded in three stages. First, all doctor-authored and AI-generated responses were provisionally coded by the LLM annotator using the same fixed, pre-specified rubric. Second, all provisional labels were reviewed by a domain expert in health communication and medical humanities, who checked each response against the rubric and corrected labels where necessary. Third, prior to final consolidation, an independent double-coding audit was conducted on a random subset of 100 consultations (300 response units), with the human coder blinded to both response source and provisional AI labels. When AI and human judgments diverged, the final label was assigned according to the human expert’s decision. All analyses reported in the main manuscript are therefore based on adjudicated labels rather than raw AI-generated annotations.

This multi-stage adjudication procedure was especially important because the same general class of system was used for response generation and for first-pass annotation; accordingly, we treated the LLM annotator as a provisional pre-coding instrument rather than as a final evaluator. Its role was to apply a fixed, human-designed coding rubric to explicit narrative-communication features across all responses. The final analytic dataset was therefore not based on these provisional labels alone. Instead, all coded outputs were subsequently reviewed through expert adjudication, and an independent double-coding audit was conducted on a random subset to assess agreement between provisional AI coding and human coding prior to adjudication, with the human coder blinded to both the provisional AI labels and response source. In addition, both AI response conditions were generated with the same base model and the same temperature setting, so the principal AI comparison reflects prompt design rather than cross-model variation. Count-based outcomes were further normalized by token length, and domain-term density was included as a control to reduce confounding from superficial differences in response length or technicality. Nevertheless, because provisional annotation was LLM-assisted, some residual measurement bias cannot be fully excluded.

All adjudicated labels from human-authored and AI-generated responses were then consolidated, with formats and metadata harmonized to ensure consistency. The resulting dataset was exported in an analysis-ready form for downstream quantitative modeling.

### 2.3. Coding and Quantitative Analysis

We operationalized narrative communication into four measurable dimensions—storytelling, empathy, personalization, and clarity—using a predefined annotation schema. Consistent with the coding framework summarized in Supplementary Table S1, storytelling was assessed through (i) narrative frequency and (ii) a binary indicator of whether the explanation reflected a patient-centered narrative framework; empathy through (i) verbal expression of emotional understanding and support and (ii) emotion-symptom linkage; personalization through (i) the overall personalization score and (ii) consideration of patient preferences; and clarity through terminology explanation frequency. This structure distinguishes the broader theoretical dimensions from the specific coded indicators used in the analyses.

To ensure comparability across texts, coding metrics were normalized by token length, and text-level characteristics were incorporated as analytic controls. Because reply length differed substantially across conditions, descriptive token-length distributions are reported in the Supplementary Materials (Supplementary Table S6) to contextualize the normalization strategy and to clarify that the prompted condition’s advantage is not reducible to simple verbosity. Domain-term density was additionally used as a proxy for medical complexity and included to account for systematic variation in language use across consultations and response types.

Quantitative analyses were conducted using count-based statistical models to compare narrative metrics across human-authored, unprompted AI, and prompted AI responses. Model uncertainty and robustness were assessed through established resampling and diagnostic procedures. Full details of model specifications, uncertainty estimation, and sensitivity analyses are provided in the Supplementary Materials.

### 2.4. Ethics and Data Governance

This study reports a secondary analysis of MedDialog-CN, a publicly available de-identified corpus of online doctor–patient consultations released for research use. We accessed the data only in the anonymized form provided by the dataset creators and did not collect new data, attempt re-identification, or link records to external sources. All analyses were conducted on non-identifiable text, and findings are reported in aggregate.

Because the work involves secondary analysis of de-identified textual data and does not entail direct interaction with human participants or the use of identifiable private information, it did not require formal institutional ethics review. All data and derived files were stored and processed on secure university infrastructure with access restricted to authorized personnel. LLM response generation and annotation were performed exclusively on de-identified inputs.

## 3. Results and Discussion

**Figure 3:**
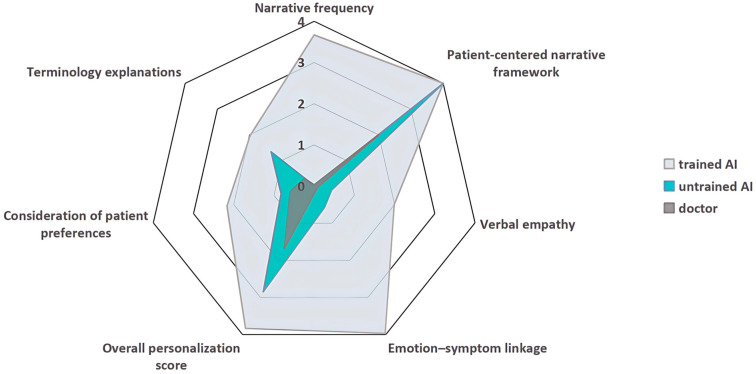
The Overall Performances across three response conditions. **Notes:** Values for Narrative frequency, Verbal empathy, Terminology explanations, and Overall personalization score are taken directly from Table 1. Values for Patient-centered narrative framework, Emotion–symptom linkage, and Consideration of patient preferences are taken from Table 2 and multiplied by 4 for plotting on a common radar scale. This rescaling is for visual comparability only and does not affect relative group differences. All raw values are reported in Table 1 and Table 2.

To evaluate whether narrative-based prompting can remediate the well-documented “communication deficit” in high-throughput care, we compared three response conditions for the same patient query: the original clinician reply, a zero-shot LLM reply, and a prompt-conditioned LLM reply. Narrative-based communication was operationalized along four dimensions: Storytelling, Empathy, Personalization, and Clarity, drawing on narrative medicine and patient-centered communication scholarship that links interpretive scaffolding and empathic attunement to trust, comprehension, and adherence-related outcomes [26,27,28]. All frequency-based indicators were operationalized as event counts per consultation and reported as the observed mean number of events per consultation across the 1000-consultation sample, whereas binary indicators were reported as proportions. This design isolates communicative form from case-mix by holding the patient input constant across the two LLM conditions, while retaining the clinician reply as the ecological baseline for real-world online consultation practice. The present findings therefore concern coded textual features of patient-facing communication rather than patient perceptions, trust, algorithm aversion, or downstream clinical outcomes, which were not directly measured in this study. Figure 3 summarizes the overall pattern across the three response conditions.

Across these metrics, the central pattern is not merely that the prompted model “scores higher,” but that prompting re-organizes the distribution of communicative labor: from sparse and selectively deployed in clinician replies to routine and highly standardized in LLM outputs, with the strongest step-change occurring after narrative prompting. For example, narrative devices were rare in clinician replies and remained uncommon under zero-shot generation, but became stable and frequent under prompting, indicating a shift from rare-event behavior to routinized narrative expression. A similar “from sparse to uniform” transition is also visible in other empathic language. These results align with prior evidence that LLM systems can produce patient-facing responses that are rated as more empathic than physicians’ replies in online advice settings [29], and with recent third-party evaluations showing that AI-generated responses may be perceived as more compassionate and responsive than human (including expert) responses even under source disclosure [30]; at the same time, clinical deployment requires safeguards beyond perceived quality.

This inversion matters because it points to a plausible mechanism behind algorithm aversion in advice contexts: what users experience as “inhuman” is often not the absence of biomedical correctness, but the absence of recognizable relational and interpretive work—naming emotions, translating technical terms, and scaffolding a coherent illness account that the patient can inhabit. Consistent with behavioral research on algorithm aversion, uptake can remain low even when algorithms perform well, because trust hinges on perceived legitimacy and relational fit rather than accuracy alone [31]. In our data, clinicians’ low rates are plausibly less a deficit of competence than a signature of structural compression: under time pressure, narrative and empathy become contingent resources deployed mainly when the interaction escalates. LLM outputs, by contrast, are time-unconstrained; zero-shot generation already imports a partially standardized “service-dialogue” template, yet remains limited in narrative explanation. Prompting then makes narrative and empathy not only more frequent but also more predictable—an affordance that could help close the interpretability gap that fuels aversion, while also raising a design question: how to preserve credibility and situational fit when relational language becomes uniform.

Importantly, the prompt advantage is not restricted to affect. It also increases informational legibility (e.g., more frequent terminology explanation), which is consistent with evidence that clarity and health literacy demands are consequential in patient understanding and use of health information [32]. However, this is also where the clinician baseline remains theoretically instructive: clinician replies may appear “lower scoring” partly because they are selectively elastic—expanding when the case becomes socially or emotionally high-stakes—whereas the prompted model expands more uniformly. That uniformity is a strength for scale and equity, but it is also the locus of the next practical frontier: calibration. A robust clinical assistant should not only produce more narrative medicine cues; it should learn when to intensify them (e.g., distress markers, prognosis anxiety, vulnerability cues) and when to compress them to avoid overload. This concern is consistent with broader work showing that patients can experience information overload in digital health contexts, with adverse effects on comprehension and intention to use self-management materials [33].

For that reason, the substantive contribution here is best framed as follows: narrative prompting demonstrates that narrative medicine skills are, to a significant extent, controllable interface properties of LLM advice rather than fixed model traits. The implication is neither “AI is more human than doctors” nor “doctors are poor communicators,” but that a lightweight prompt can reallocate communicative resources in a way that may be difficult to sustain consistently in high-volume human practice—and that the remaining challenge is to replace uniform “always-on” humanistic talk with context-sensitive dosing that preserves both authenticity and patient safety. Table 1 summarizes the mean values of the frequency-based communication metrics across the three response conditions.

**Table 1:** Mean values of the frequency-based communication metrics across the three models.

	Doctor	Unprompted AI	Prompted AI
Narrative frequency	0.031	0.012	3.669
Verbal empathy	0.092	0.434	1.99
Terminology explanations	0.301	1.337	1.981
Overall personalization score	1.674	2.846	3.833

Table 2 reports the prevalence of the binary communication behaviors across the three response conditions.

**Table 2:** Prevalence of the binary communication behaviors across the three models.

	Doctor	Unprompted AI	Prompted AI
Emotion–symptom linkage	0.026	0.144	0.991
Consideration of patient preferences	0.15	0.206	0.542
Patient-centered narrative framework	0.499	1	1

### 3.1. Storytelling (Narrative-Device Density) Across Response Conditions

Narrative medicine positions clinical communication as more than information transfer: it depends on how clinicians recognize and respond to patients’ accounts as lived experience, thereby shaping trust and interpretability [23,26]. In health communication, narrative and figurative formats can shift attention, comprehension, and persuasion via mechanisms distinct from expository information [34,35]. At the same time, figurative framing is not a stylistic “add-on.” Even subtle metaphor cues can alter reasoning, and dominant public-health metaphors (e.g., war/battle framings) may undermine perceived agency or prevention intentions in some contexts [36,37]. For this reason, we treat “storytelling skill”—operationalized as the use of analogies, metaphors, and brief illustrative mini-stories—as a calibratable resource: it may make biomedical concepts graspable, yet it can also amplify rhetorical force and shape inference in unintended ways.

Using the updated rare-event modeling strategy (Poisson models with token-length offsets; bootstrap confidence intervals), storytelling devices were scarce in clinician replies and in the unprompted LLM condition, but became a stable default under narrative prompting. Specifically, the estimated narrative-device rate was 0.031 for clinicians and 0.012 for unprompted AI (≈12 narrative instances per 1000 dialogues; bootstrap 95% CI [0.006, 0.019]), consistent with a rare-event regime. In contrast, the narrative-prompted condition exhibited a mean of 3.669 narrative instances per dialogue (bootstrap 95% CI [3.576, 3.762]) with slight underdispersion, indicating both markedly higher density and more uniform delivery across cases. These contrasts imply that “storytelling” in this dataset is structurally constrained for clinicians (and largely absent in zero-shot generation), yet readily inducible—and quickly standardized—via a short narrative prompt.

A qualitative audit of the clinician narratives helps interpret why the clinician condition remains in a rare-event regime despite the known pedagogical value of metaphor and narrative. The physician corpus contains only a small set of narrative utterances, and they appear targeted rather than routine: most draw on everyday experiential domains (food, clothing, balloons, insects, household objects), which plausibly supports mental simulation and recall by translating technical abstractions into concrete imagery. This restricted but “vernacular” repertoire is consistent with broader evidence that metaphors can aid understanding—but only when the chosen frame fits the patient’s interpretive needs and does not import harmful entailments (e.g., fatalistic or blame-laden implications) [37,38,39]. Put differently, the clinician pattern looks less like an absence of communicative competence than a scarcity of opportunity: figurative elaboration is reserved for interactional moments where the informational and affective stakes rise, rather than being deployed as a default explanatory style.

Two further features of the clinician narratives matter for interpretation and for designing “humanistic” AI outputs. First, the narratives are often interactionally contingent: they tend to surface after multiple follow-up questions or in moments of heightened urgency/anxiety, suggesting that clinicians use narrative elaboration as a form of escalation—activated when routine biomedical shorthand fails to secure alignment. Second, some narrative turns are culturally anchored (e.g., proverbs; occasional self-disclosure), which can function as rapport work in Chinese consultations but also creates context-sensitivity and potential variability in how patients interpret the frame. This is precisely where a “more narrative” AI is not automatically a “better” AI: increasing narrative density without attending to situational fit risks producing over-elaboration, misplaced reassurance, or frames that inadvertently reduce agency [37,38].

Against that baseline, the unprompted LLM’s low narrative density is diagnostically important. Despite operating free of time pressure, the model produces few figurative turns, and these are typically templatic, predominantly descriptive analogies that foreground surface similarity but do not reliably perform the interpersonal work often achieved by well-timed clinician metaphors (e.g., validation, containment of uncertainty, and actionable orientation). This pattern aligns with the broader point that narrative effects are not driven by “more text” or “more metaphors” per se, but by how messages guide interpretation, agency, and decision-relevant inference [34,36,40]. It also converges with controlled comparisons of human and LLM storytelling under identical prompts, which show that default LLM narratives tend to be more standardized and less imaginative than human-authored stories [41].

The narrative-prompted condition, by contrast, shows what is most novel and most publication-relevant about the present design: minimal instruction shifts storytelling from a rare, contingent device to a predictable explanatory default, producing a ~two-orders-of-magnitude increase in narrative density (3.669 per dialogue vs. ≤0.031 in the other conditions). This gain is plausibly valuable insofar as narrative devices can increase engagement, comprehension, and recall—especially for complex risk information—yet the literature also warns that some metaphor families (notably “battle” framings) can reduce prevention intentions or induce fatalism when mismatched to the task or the patient’s perceived control [37,38,39]. The design implication is therefore not simply “prompt for more storytelling,” but “prompt for calibrated storytelling”: systems should be engineered to (i) detect when figurative elaboration is needed for alignment and (ii) constrain frames that carry known undesirable entailments. One practical route is to couple narrative prompting with lightweight safety constraints (e.g., disallow high-risk metaphor families in prevention contexts; require explicit uncertainty statements when evidence is limited) and to condition narrative elaboration on linguistic markers of confusion, anxiety, or repeated questioning—mirroring the contingency observed in clinician behavior.

### 3.2. Patient-Centered Narrative Framework Across Response Conditions

Patient-centeredness is commonly defined as a shift from a disease-centered account toward the patient-as-person, including attention to goals, constraints, and the sharing of power and responsibility in care decisions [2]. Related work on shared decision making (SDM) likewise emphasizes partnership, option awareness, and the incorporation of patient preferences into clinical communication [3,42,43]. In the present study, however, we did not operationalize the full construct of patient-centeredness or SDM. Rather, consistent with Supplementary Table S1, we coded a narrower binary indicator: whether the explanation reflected a patient-centered narrative framework. This indicator was marked as present when the explanation was organized around the patient as the experiential center of the account, rather than presented only as decontextualized biomedical information. It should therefore be interpreted as a storytelling-related indicator within the broader narrative communication framework, rather than as a comprehensive measure of patient-centeredness or shared decision making.

Quantitatively, clinicians used a patient-centered narrative framework in 49.9% of replies (mean proportion = 0.499, 95% CI [0.469, 0.530]). In contrast, the unprompted LLM met the binary criterion in essentially all replies (proportion = 1.000, 95% CI ≈ [0.996, 1.000]), and this ceiling-level framing persisted under narrative prompting (proportion = 1.000). On its face, the result suggests that patient-centered narrative framework—at least as a detectable textual feature—is more reliably produced by the model than by time-pressured clinicians. The more consequential question, however, is what kind of “patient-centeredness” is being operationalized when a system reaches saturation.

Qualitatively, the clinician corpus illustrates why a 50% rate can still correspond to thin patient-centeredness. Many clinician replies contained a single contextual anchor (e.g., reproductive plans or scheduling constraints) but then closed down the interaction through directive sequencing (brief imperatives, minimal option comparison, little preference elicitation). This pattern is compatible with qualitative descriptions of outpatient encounters in China where high throughput and institutional routines compress opportunities for extended negotiation, yielding an efficient but clinician-led interactional trajectory [44]. The key point is not that clinicians never frame advice around patients’ lives, but that life-world anchoring often functions as justification for a recommendation rather than an opening for deliberation—a distinction central to patient-centeredness models that explicitly include sharing power and responsibility [2,43].

By contrast, the unprompted LLM’s ceiling performance appears to be driven by a procedural script that is easy for a binary code to “reward”: a polite acknowledgement, a generic anchoring move (“in daily life…/in your routine…”), and a recommendation. This yields high reproducibility but limited responsiveness to stated preferences as negotiable constraints—consistent with broader concerns that LLMs, without steering, tend toward courteous, templated counselling that may remain weak on preference-sensitive trade-offs and local feasibility. The risk is that patient-centered narrative framework is reduced to a surface pattern of personal address and contextualization rather than functioning as a genuinely deliberative communicative move [42]. In addition, saturation raises a readability concern: consistently long, multi-part counselling can increase cognitive burden for users with limited health literacy or those already experiencing informational overload in digital health contexts [33].

Narrative prompting, in turn, changes the functional texture of framing even when the binary rate cannot increase further. Prompted replies more consistently convert anchoring into conditional pathways (“if you prefer…”, “if access is limited…”) and explicit option structuring (summaries of alternatives and recommended next steps), aligning more closely with SDM’s “option talk” and “decision talk” moves [42]. This is where the practical promise lies: not merely that AI can sound patient-centered, but that it can standardize an option-oriented structure that is often sacrificed under clinical time constraints [44]. At the same time, prompt-enabled framing introduces governance trade-offs: if options are presented without sensitivity to local formularies, service availability, or clinical thresholds, patients may experience “false choice” or later contradiction by human clinicians, undermining trust—especially in preference-sensitive decisions where SDM depends on realism and context specificity [3,42]. For this reason, the ceiling result should be interpreted as reliability of a communicative form, not proof of superior decision quality; future iterations should couple framing prompts with locality constraints (e.g., setting-specific availability flags) and calibrated brevity to reduce overload.

### 3.3. Empathic Communication: Explicit Support and Emotion Validation Across Response Conditions

Empathy in clinical communication is commonly operationalized as the ability to recognize a patient’s emotional cues and to respond with language that conveys understanding, respect, and support. Across modalities, empathic communication has been associated with improved satisfaction, reduced anxiety, better disclosure, and measurable clinical outcomes [4,27,45]. In text-based consultations, where nonverbal reassurance is unavailable, empathic meaning must be conveyed almost entirely through wording and sequencing, especially through (i) explicit supportive statements (e.g., acknowledging worry and offering reassurance) and (ii) validation or normalization that frames the patient’s emotion as understandable and legitimate. Because these two moves can diverge in practice (support without validation, or a single high-impact validation without sustained support), we analyze them as complementary components within the broader empathy dimension.

#### 3.3.1. Verbal Expressions of Emotional Understanding and Support

**Figure 4:**
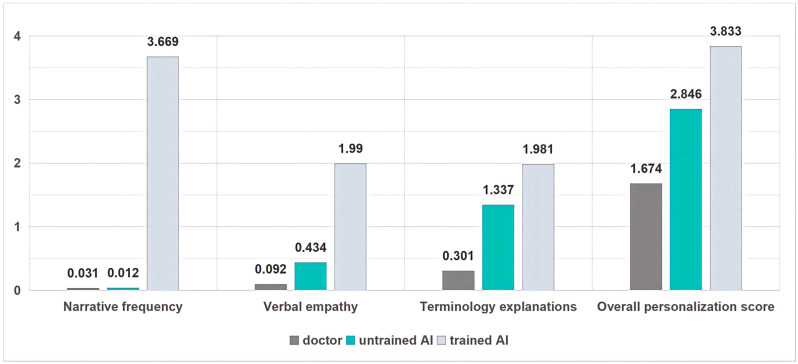
Radar plot of the frequency-based communication metrics across three models.

Figure 4 shows a pronounced three-condition gradient in the frequency of explicit empathic-support statements. Physician replies exhibit a near-floor distribution: the mean count is 0.092 per reply, and 93.2% of physician replies contain no explicit supportive-empathy statement at all. This pattern is consistent with long-standing findings that high-throughput clinical environments tend to prioritize biomedical task completion over affective talk, even when clinicians endorse empathy as a professional value [27,46].

The unprompted LLM condition moves meaningfully above this baseline but remains sparse in absolute terms: the mean is 0.434, with 68.8% of replies still containing zero supportive-empathy statements. Qualitatively, when such statements appear, they frequently cluster in formulaic boundary positions (opening or closing lines), functioning as interactional “headers/footers” rather than as an ongoing responsiveness to the patient’s unfolding concerns. This matters because empathy is not simply the presence of a sympathetic clause; its perceived authenticity often depends on contingency—whether the clinician (or system) demonstrably tracks what the patient is worried about, and returns to it at decision-relevant moments [27].

Under narrative prompting, the distribution is qualitatively transformed. The mean rises to 1.99 supportive-empathy statements per reply—an order-of-magnitude shift relative to physicians—and the pattern no longer clusters at zero. Importantly, this gain is not merely “more warmth.” In many prompted replies, empathic language is interleaved with explanation and advice, approximating established communication teaching heuristics that encourage clinicians to name, understand, or support emotions and then return to information exchange [47]. The methodological implication is consequential: in asynchronous, text-only environments, a short prompt can function as a low-cost “empathy scaffold,” shifting empathic talk from sporadic ornamentation to a routinized design feature.

At the same time, the prompted condition’s consistency raises a known design risk: when empathic moves become highly standardized, they may drift toward perceived “scriptedness,” especially if repeated lexical templates recur across consultations. Relational-agent research and evaluations of automated supportive dialogue systems repeatedly note that perceived sincerity depends on variation and context-linking, not just presence [27,48]. For an applied system, the challenge is therefore not to add empathy (prompting already does) but to differentiate empathy—lexically, temporally (when it appears), and conditionally (what triggers it).

#### 3.3.2. Validation Through Explicit Emotion-Symptom Linage

To move beyond generic reassurance, we further assess whether replies explicitly link the patient’s emotion to the situation described (e.g., validating worry as understandable given symptoms, uncertainty, waiting, or perceived risk). This “emotion–symptom linkage” captures a stronger form of validation because it displays interpretive uptake: it shows the responder has not only noticed emotion but also recognized why it is reasonable in context.

Here the three conditions diverge even more sharply. Physicians almost never make this link (2.6%; 95% CI 1.8–3.7%), and the unprompted model—while higher—remains limited (14.4%; 95% CI 12.4–16.7%). Under narrative prompting, however, the probability becomes near-universal (99.1%; 95% CI 98.3–99.5%).

Analytically, this is the crucial distinction between polite sympathy and contextually grounded validation. The prompted model does not merely say “I understand”; it typically makes the understanding accountable to the patient’s predicament (uncertainty, fear of progression, discomfort, or downstream consequences). This is precisely the move that communication scholarship links to perceived partnership and respect, because it treats emotion as epistemically legitimate rather than as noise around the biomedical problem [27,46]. Yet the same caveat applies: a validation move repeated with minimal lexical or situational differentiation can become a “reassurance stamp,” risking habituation effects and credibility loss over time.

Taken together, the empathy findings refine the manuscript’s broader argument about “humanistic AI.” The prompt does not make the model feel empathy; rather, it reliably induces a set of interactional practices that patients may experience as empathic—explicit support, validation, and context-linking—at a coverage level that resource-constrained human services rarely sustain. The next scientific step is not simply to celebrate this coverage, but to test patient-perceived authenticity, cognitive burden, and safety interactions (e.g., whether empathic validation co-occurs with appropriate uncertainty and triage advice) under realistic deployment constraints.

### 3.4. Personalization: Contextual Tailoring and Preference-Sensitive Guidance Across Response Conditions

**Figure 5:**
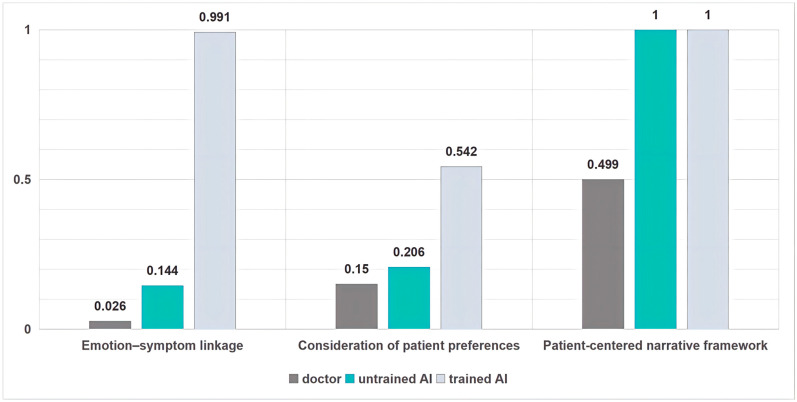
Radar plot of the binary communication behaviors across the three response conditions.

Personalization is a core pillar of patient-centred communication: it adapts medical advice to the patient’s lived constraints (work rhythms, family responsibilities, access barriers) and, at a stronger level, incorporates the patient’s stated preferences into decision-relevant guidance. Tailored health communication has been repeatedly linked to improved relevance and message processing, and in some contexts to better adherence-related outcomes compared with generic advice [49,50]. In shared decision-making (SDM) frameworks, personalization becomes ethically and practically consequential when it supports deliberation—helping patients weigh options in light of what they value—rather than merely decorating advice with contextual flourishes [2,42]. Accordingly, we evaluate personalization along two complementary components: (i) overall contextual tailoring (a graded score) and (ii) explicit consideration of patient preferences (binary). Figure 5 shows the prevalence of the binary communication behaviors across the three response conditions.

#### 3.4.1. Contextual Tailoring

Using the updated quantitative framework, clinicians achieved a mean overall personalization score of 1.674 (bootstrap 95% CI [1.612, 1.738]). Unprompted AI was higher at 2.846 ([2.767, 2.922]), while the narrative-prompted condition was highest at 3.833 ([3.775, 3.895]). This gradient matters not only as a difference in magnitude but as a difference in interactional style: prompting appears to convert personalization from a selective resource into a default explanatory architecture.

Qualitatively, this shift is best interpreted through what personalization is doing in each condition. Clinicians’ personalization often functions as constraint-spotting—identifying a single barrier most likely to derail feasibility (e.g., work schedule, distance, cost) and adjusting around it. This is efficient and may be appropriate under extreme throughput constraints, but it can also appear limited as a form of patient-centered communication when the selected barrier is not the one most salient to the patient’s concern. By contrast, unprompted AI tends to produce scripted contextualization (routine anchoring to “daily life”), which can satisfy a rubric but remain shallow in feasibility checking or contingency planning—an issue consistent with broader cautions that generic personalization can feel relevant while still being practically brittle [50]. Prompted AI, however, more often produces anticipatory personalization: it makes advice conditional (“if-then”) and pre-empts logistical barriers. The substantive contribution is therefore not simply “more tailoring,” but a different logic of tailoring: from single-constraint adjustment toward branching contingency planning.

This improvement nonetheless introduces a predictable cost: richer tailoring can increase message length and structural complexity, which may overload readers with limited health literacy. Evidence syntheses in health literacy show that comprehension and effective use of health information are strongly shaped by readability and cognitive demands [32]. Cognitive load theory similarly warns that added complexity can yield diminishing returns once working-memory limits are exceeded [51]. Thus, the design target is not maximal personalization, but right-sized personalization: enough to make advice feasible; not so much that it becomes unscannable.

#### 3.4.2. Preference-Sensitive Guidance

Preference incorporation is conceptually distinct from contextual tailoring. A response may tailor advice to constraints while still retaining unilateral decisional authority. Here the updated results show a more modest, but practically important, prompt effect. Clinicians incorporated patient preferences in 15.0% of replies (bootstrap 95% CI [0.128, 0.172]). Unprompted AI was slightly higher at 20.6% ([0.181, 0.231]). Prompted AI increased preference-sensitive guidance to 54.2% ([0.512, 0.573]).

Interpreted through SDM theory, this pattern suggests that the narrative prompt is not only adding warmth or detail; it partially shifts the interactional stance from “recommend” toward “negotiate.” This aligns with SDM models that emphasize eliciting values and offering options in a way that supports deliberation [42]. However, the fact that prompted AI remains far from ceiling on preference incorporation (≈54%) is analytically useful: it indicates a harder problem than generic contextualization. Preference-sensitive guidance requires recognising that a preference has been expressed, interpreting it as decision-relevant, and then modifying advice accordingly—an inferential chain that can fail even when a system is fluent and empathic.

Taken together, the results suggest a division of labour. Clinicians’ personalization is often selective and efficiency-bounded; unprompted AI provides consistent but generic contextualization; prompting yields high and structured tailoring while also increasing preference sensitivity. The remaining deployment challenge is governance: AI-generated branching plans can become “false choice” if they are not constrained by local service availability, formularies, or thresholds, potentially eroding trust when later contradicted by clinical reality [32,42]. The pragmatic next step is therefore an architecture that combines (i) context-adaptive prompting (to calibrate length and depth), with (ii) locality constraints (to avoid infeasible options), and (iii) health-literacy aware formatting (to preserve usability under cognitive load).

### 3.5. Clarity: Closing the Explanation Gap in Patient-Facing Advice

Clarity in clinician–patient communication is not achieved by eliminating medical terminology; it is achieved when necessary terms are translated into patient-usable meaning—briefly, accurately, and in a way that supports action. This matters because limited health literacy is consistently associated with poorer understanding and worse health outcomes, and because unclarified jargon can produce “silent misunderstanding” even when patients appear to agree [32,52]. In digital consultations, where patients cannot easily interrupt to request clarification, the practical risk is an explanation gap: terms are introduced without an adjacent gloss that connects meaning to implication.

The updated results show that this explanation gap is structurally common in clinician replies but markedly reduced in both AI conditions, especially after prompting. Clinicians provided on average 0.301 terminology explanations per reply (bootstrap 95% CI [0.259, 0.348]), and 81.1% of clinician replies contained no terminology explanation at all (Wilson 95% CI [0.786, 0.834]). Unprompted AI offered substantially more explanations (1.337, 95% CI [1.256, 1.421]) and reduced the “no-explanation” proportion to 38.3% (Wilson 95% CI [0.353, 0.414]). With the narrative prompt, explanation density increased further to 1.981 (95% CI [1.901, 2.061]), while the share of replies offering no explanation fell to 15.3% (Wilson 95% CI [0.132, 0.177]). The substantive shift is therefore not only “more explanation,” but a change in what becomes normal: under prompting, providing at least some clarification becomes the default rather than a contingent response to patient confusion.

A closer interpretive reading suggests that the three conditions represent distinct “clarity regimes.” Clinician replies often rely on institutional shorthand: terminology is embedded in a directive or diagnostic label and allowed to stand on professional authority, with unpacking supplied—if at all—only when a patient displays non-understanding. This economy is interactionally rational under throughput pressure, but it externalizes interpretive labour to patients and creates the conditions that health-literacy research repeatedly identifies as hazardous: patients may comply without comprehension, or they may misinterpret the relevance and urgency of advice [32,53]. Zero-shot AI partly closes the gap by defaulting to generic definitional glosses, which lowers the probability that a term is left entirely unexplained. Yet, without steering, these glosses can remain weakly action-linked—defining what a term “is” without translating what it means for monitoring, thresholds, or next steps. Prompting shifts the model toward a more clinically useful sequencing: explanation is more often delivered as term → plain-language paraphrase → practical implication, effectively turning clarification into a small scaffold that supports actionability rather than mere comprehension.

At the same time, clarity is not a monotonic “more is better” variable. Over-explanation can increase cognitive load and overwhelm readers, particularly those with limited health literacy [32,51]. The design implication, therefore, is calibration rather than maximal verbosity: AI systems should be engineered to identify high-impact terms that require glossing, provide brief explanations linked to actionable meaning, and adjust depth to cues of confusion, anxiety, or repeated questioning. In this sense, prompting demonstrates that clarity is highly controllable at the interface level, while the remaining challenge is governance: ensuring that added explanatory text reduces misunderstanding without creating overload or masking uncertainty.

### 3.6. Limitations

Several limits on external validity should be noted. In addition, because reliable consultation-level disease or specialty stratification was not available in the released corpus, we could not test directly whether the effects of narrative prompting varied across clinical domains with different empathic or explanatory demands. First, this study is based on Chinese, asynchronous, text-based online consultations drawn from a single platform, so its strongest generalizability is to similar digital advice environments rather than to healthcare communication in general. Second, although the coded communication functions examined here—including empathy, explanation, personalization, and the presence of a patient-centered narrative framework—are broadly relevant across healthcare systems, their linguistic realization is not culturally neutral. The ways in which support, directness, metaphor, reassurance, and preference-sensitive guidance are expressed and interpreted may vary across languages, sociocultural contexts, and platform norms. Third, text-only consultations differ from face-to-face and synchronous video encounters because they lack prosody, gesture, and other nonverbal resources; in such settings, explicit verbal empathy and terminology explanation may carry greater interactional weight than they would in offline care. For these reasons, the present findings are best interpreted as evidence for a transportable design principle: narrative prompting can increase relational and interpretive scaffolding in patient-facing AI responses, but the same magnitude or stylistic profile should not be assumed across languages, specialties, or clinical modalities. Future work should therefore test this approach in MedDialog-EN and other multilingual datasets, as well as in specialty-specific, synchronous, and offline consultation settings.

## 4. Conclusions

This study examined whether a brief narrative prompt could alter the communicative profile of AI-generated medical advice in Chinese asynchronous online consultations. Using 1000 consultations, we compared clinicians’ original replies with zero-shot and narrative-prompted LLM responses on four communication dimensions—storytelling, empathy, personalization, and clarity—while holding the patient query constant across AI conditions.

The main finding is not simply that the prompted model performed better on several measures. Rather, the prompt changed which communicative features appeared routinely in the response. Practices that were sparse and selectively deployed in clinician replies—figurative explanation, explicit emotional support, context-linked validation, structured tailoring, and proactive term glossing—became markedly more common under prompting. These results suggest that several relational and interpretive features of patient-facing communication can be shaped through prompt design.

The clinician baseline remains instructive. Whereas the model tends to standardize warmth and elaboration, clinicians appear to reserve extended narrative or emotional labor for moments of interactional pressure. The next step is therefore not maximal humanistic language, but context-sensitive calibration: intensifying interpretive scaffolding when patients display confusion, distress, or preference-sensitive uncertainty, while preserving brevity and triage discipline when risk and time constraints require it.

The study’s contribution is thus a context-bounded design claim with ethical stakes: in Chinese asynchronous, text-based online consultations, narrative prompting may improve the coded patient-facing communication profile of AI-generated responses, but it also raises governance questions about credibility, local feasibility, cognitive load, and the risk of scripted empathy. Whether comparable gains extend to other languages, specialties, and clinical modalities requires direct validation. Future work should test patient-facing outcomes such as comprehension, trust calibration, and decisional conflict, develop adaptive prompting tied to patient cues, and incorporate locality constraints so that generated options remain clinically and logistically realistic.

Overall, a brief narrative instruction can move an LLM beyond efficient explanation toward a richer profile of coded relational and interpretive communication features. The remaining challenge is to ensure that these prompt-induced features remain calibrated, situated, and safe when used at scale.

## Data Availability

The consultation data analyzed in this study are from MedDialog-CN, a publicly available, de-identified dataset (see Reference [25]). The derived data generated for this study (LLM outputs and adjudicated annotation labels) and analysis scripts are available from the corresponding author upon reasonable request due to the large file size and responsible-release considerations (The derived corpus includes large-scale LLM-generated medical advice, and unrestricted redistribution could facilitate harmful reuse (e.g., repackaging as ready-to-use diagnostic guidance without appropriate oversight, or reusing the corpus to optimize prompts that encourage overconfident medical output). In addition, although the source dataset is de-identified, releasing large volumes of text combined with metadata/annotations may increase residual re-identification risk through aggregation and linkage with external traces (the “mosaic effect”). Finally, online consultation texts may contain sensitive health information; even when anonymized, broad dissemination of full outputs and associated annotations raises ethical concerns unless release is tightly scoped and controlled) related to redistribution of patient-facing medical advice text.

## Supplementary Materials

The following supporting information can be downloaded at: https://www.mdpi.com/article/10.3390/healthcare14081015/s1.
