# Supplementary material for: Unlikely Storyteller: Leveraging Narrative-Based Communication in LLM-Generated Medical Advice
Source: Healthcare (Basel). 2026 Apr 13;14(8):1015. doi: 10.3390/healthcare14081015 (PMC13116779; doi:10.3390/healthcare14081015)
Supplement: Supplementary file 1 [file healthcare-14-01015-s001.zip › healthcare-4215930-supplementary.pdf]

Supporting Information

of

Unlikely storyteller: Leveraging Narrative-Based Communication in LLM-Generated Medical Advice

Table of Contents

Table of Contents 2
1 Data Collection and Preparation 3
1.1 Session segmentation and speaker identification 3
1.2 Word segmentation and medical term identification 3
1.3 Data storage and ethics 3
2 LLM-assisted generation and adjudication 3
2.1 LLM-assisted response generation 3
2.2 Coding schema design 9
2.3 Normalization and analytic controls 11
3 Statistical Modeling and Inference Framework 11
3.1 Statistical modeling framework 11
3.2 Uncertainty estimation and confidence intervals 12
3.3 Detailed Quantitative Analysis of Narrative Communication 13
3.3.1 Storytelling 14
3.3.2 Empathy 16
3.3.3 Personalization 17
3.3.4 Clarity 18
3.4 Model diagnostics and robustness checks 19

1 Data Collection and Preparation

1.1 Session segmentation and speaker identification

Raw consultation logs were provided as concatenated text files containing multiple dialogue sessions. These files were programmatically split into individual consultation sessions based on session delimiters. Within each session, speaker turns were identified using role tags embedded in the original corpus metadata. Consultations containing fewer than two complete doctor–patient exchanges were excluded to ensure sufficient interactional context for narrative coding.

A basic quality filter was applied to remove malformed or incomplete sessions (e.g., truncated conversations or missing speaker roles). The retained sessions were stored in a structured text format for downstream processing.

1.2 Word segmentation and medical term identification

Chinese word segmentation was performed using the Jieba tokenizer with its default configuration. To support subsequent analytic controls, medical domain terms were identified using an LLM-assisted tagging procedure, in which candidate medical entities were flagged based on domain-specific lexical patterns and contextual cues. The output of this process was used solely for text-level characterization rather than annotation or outcome coding.

Based on the tokenized text and identified medical terms, we computed a domain-term density metric for each consultation, defined as the proportion of medical domain tokens relative to total token count. This metric served as a proxy for medical complexity and was later incorporated as a control variable in statistical models.

1.3 Data storage and ethics

The primary corpus used in this study is an open-source clinical dialogue database whose curators had already removed direct identifiers (e.g., physician and patient names). Prior to analysis we applied additional de-identification to location and institutional metadata (hospital and department) to further reduce re-identification risk. As a result, all records analyzed were fully de-identified and no personally identifiable information was retained.

All intermediate and final text files were stored in a secure research environment with access restricted to the research team. Processed datasets were versioned to ensure traceability across preprocessing, annotation, and analysis stages.

2 LLM-assisted generation and adjudication

2.1 LLM-assisted response generation

For each consultation, the patient query was first summarized using a large language model (LLM) to reduce irrelevant details while preserving clinical intent. Based on this summary, two types of AI responses were generated:

(1) a zero-shot condition, in which the LLM produced a response without any task-specific instructions;

(2) a prompted condition, in which the LLM was provided with a detailed narrative prompt specifying response length, tone, and communicative goals.

Both AI-generated replies were produced independently for each query. All generated responses were retained verbatim and paired with the original doctor response from the consultation.

Unprompted AI Model

The unprompted AI model is ChatGPT-o3-mini operating without specialized conditioning or instructions related to narrative medicine. Patient queries from the MedDialog-CN dataset are input verbatim into ChatGPT-o3-mini, and the resulting responses are collected and analyzed with no additional modifications. This setup aims to replicate how an AI model might respond in real-world scenarios without any prompts guiding it toward empathy, storytelling, clarity, or personalization—core elements of narrative-based communication. Although the unprompted AI is likely to provide factually correct and concise medical information, it may lack the emotional depth, contextual relevance, and user-friendly explanations required to fully engage patients on a humanistic level.

Prompted AI Model

By contrast, the prompted AI model employs ChatGPT-o3-mini conditioned with prompt engineering that emphasizes narrative-based communication skills. While not fine-tuned on any specific narrative medicine dataset, this approach uses specially designed prompts to encourage empathy, storytelling, personalization, and clarity in the AI's responses. Such conditioning aims to align the AI's response more closely with narrative medicine principles, bringing it nearer to the humanistic qualities often missing in automated systems. As a result, the prompted AI seeks to simulate deeper emotional rapport, offer more vivid illustrations, and deliver explanations attuned to individual patient contexts—all of which can foster enhanced patient trust and engagement.

To train the AI for this condition, each patient query is paired with a set of narrative-based prompts and evaluation methods that instruct the AI to respond using the following techniques:

Table S1. Narrative-based prompts and evaluation methods

**Skill**	**Definition**	**ExamplePrompt**	**Evaluationmethod**
Storytelling The AI is prompted to explain the medical condition	Doctors explain medical concepts through narration, analogy, or	"Explain medical concepts using analogies or metaphors that are easy for patients to	Count the number of narrative elements used by doctors, such as metaphors, stories, analogies, etc. The

Skill	Definition	ExamplePrompt	Evaluationmethod
or advice using relatable analogies or narratives that align with the patient's personal context.	metaphor	understand (such as: 'Your heart is like a water pump, blood vessels are like water pipes, blockage is like rusting of water pipes')"	specific standard is the number of narrative elements used.
	Doctors use a patient-centered narrative framework for explanation	" Narrate the development of the disease with the patient as the protagonist (such as: 'When you stay up late, your immune system is like a soldier who stays up late, and your combat effectiveness decreases')."	Determine whether the explanation reflects a patient-centered narrative framework. The quantitative standard is binary judgment, with existence being 1 and non existence being 0.
Empathy<br>The AI is instructed to acknowledge the emotional state of the patient and respond with empathy.	Doctors express their understanding and support for patients' emotions through verbal communication	"When the patient describes discomfort, explicitly respond emotionally (such as: 'Fever for three days does indeed make people anxious, I understand your concern')."	Calculate the frequency of doctors using statements that express empathy, such as' I understand how you feel '. The standard is the frequency of expressing empathy.
	Doctors confirm patients' emotional experiences and correlate their symptoms	"Proactively associating emotions with symptoms (such as: 'The stress you mentioned may exacerbate stomach pain, which is a common bodily response')."	Observe whether the doctor asks or confirms the patient's emotional state and whether it is related to the patient's symptoms or condition. The quantitative standard is binary judgment, with existence being 1 and non existence being 0.
Personalization<br>The AI is guided to tailor the advice to the	Doctors provide customized medical advice based on the	"Customize recommendations based on the patient's	Based on factors such as the patient's lifestyle, occupation, and family

Skill	Definition	ExamplePrompt	Evaluationmethod
specific circumstances of the patient, such as their lifestyle or family responsibilities.	patient's specific situation and living environment.	occupation/habits (such as: 'As a programmer who needs to sit for a long time, it is recommended to get up and stretch every hour')."	background, a Likert scale is used for quantitative evaluation (1 point for general recommendations, 5 points for highly personalized recommendations).
	Doctors consider patients' personal preferences when providing advice	"When patients express preferences, clearly adopt and adjust suggestions (such as: 'If you like swimming, it's more suitable for your knees than running')."	Determine whether customized recommendations that consider patient preferences are included. The quantitative standard is binary judgment, with existence being 1 and non existence being 0.
Clarity<br>The AI is directed to ensure that its response is clear, avoiding medical jargon, and providing simple explanations for any technical terms.	Interpretation frequency of medical terms by doctors	"When referring to medical terminology for the first time, use parentheses to supplement the vernacular explanation (such as: 'CT (a multi-slice scanning X-ray technique) needs to be examined')."	Count the number of times doctors explain medical terminology to measure clarity in communication.

Table S2. Decoding parameters for response generation

Parameter	Value
Model version	o3-mini
Temperature	1
top_p	1
max_tokens	100,000
frequency_penalty	0
presence_penalty	0
System prompt	"You are a helpful assistant."
User prompt	See Supplementary Table S3

The first-pass annotation stage also used GPT-o3-mini with a fixed system prompt and a fixed annotation prompt applied uniformly across all response sources; temperature was fixed at 1 throughout this stage. The exact user prompts used for generation and annotation are reproduced in Supplementary Table S3.

Table S3. Prompts used for generation and annotation tasks

Condition	User prompt
Unprompted AI	To improve the effectiveness of doctor-patient communication, assume you are a doctor and respond to the patient's consultation above.
Prompted AI	To improve the effectiveness of doctor-patient communication, assume you are a doctor and respond to the patient's consultation in a way that meets the following evaluation criteria as closely as possible:<br>Criteria:<br>1. Storytelling<br>1.1 Doctors explain medical concepts through narration, analogy, or metaphor.<br>Description: Explain medical concepts using analogies or metaphors that are easy for patients to understand (such as: 'Your heart is like a water pump, blood vessels are like water pipes, blockage is like rusting of water pipes').<br>Evaluation method: Count the number of narrative elements used by doctors, such as metaphors, stories, analogies, etc. The specific standard is the number of narrative elements used.<br>1.2 Doctors use a patient-centered narrative framework for explanation.<br>Description: Narrate the development of the disease with the patient as the protagonist (e.g., "When you stay up late, your immune system is like a soldier who stays up late, and your combat effectiveness decreases").<br>Evaluation method: Determine whether the explanation reflects a patient-centered narrative framework. The quantitative standard is binary judgment, with existence being 1 and non-existence being 0.<br>2. Empathy<br>2.1 Doctors express their understanding and support for patients' emotions through verbal communication.<br>Description: When the patient describes discomfort, explicitly respond emotionally (e.g., "Having a fever for three days does indeed make people anxious; I understand your concern").<br>Evaluation method: Calculate the frequency of doctors using statements that express empathy, such as "I understand how you feel". The standard is the frequency of expressing empathy.<br>2.2 Doctors confirm patients' emotional experiences and correlate them with their symptoms.<br>Description: Proactively associate emotions with symptoms (e.g., "The stress you mentioned may exacerbate stomach pain; this is a common bodily response").<br>Evaluation method: Observe whether the doctor asks about or confirms the patient's emotional state and whether it is connected to the patient's symptoms or condition. The quantitative standard is binary judgment, with existence being 1 and non-existence being 0.<br>3. Personalization<br>3.1 Doctors provide customized medical advice based on the patient's specific situation and living environment.<br>Description: Customize recommendations based on the patient's occupation/habits (such as: 'As a programmer who needs to sit for a long time, it is recommended to get up and stretch every hour').

	Evaluation method: Based on factors such as the patient's lifestyle, occupation, and family background, a Likert scale is used for quantitative evaluation (1 point for general recommendations, 5 points for highly personalized recommendations).<br>3.2 Doctors consider patients' personal preferences when providing advice.<br>Description: When patients express preferences, clearly adopt and adjust suggestions (e.g., "Since you enjoy swimming, that is more suitable for your knees than running").<br>Evaluation method: Determine whether customized recommendations that consider patient preferences are included. The quantitative standard is binary judgment, with existence being 1 and non-existence being 0.<br>4. Clarity<br>4.1 Doctors explain medical terminology when it is first introduced.<br>Description: When referring to medical terminology for the first time, supplement with a plain-language explanation in parentheses (e.g., "You need a CT scan (a type of X-ray technique that takes multi-layered images)").<br>Evaluation method: Count the number of times doctors explain medical terminology to measure clarity in communication.
Annotation	The above is a conversation record of a doctor's consultation response to a patient. Please evaluate the doctor's communication skills during the interaction based on the following criteria:<br>1.Storytelling<br>1.1 The doctor uses narratives, analogies, or metaphors to explain medical concepts.<br>Evaluation method: Count the number of narrative elements used by the doctor, such as metaphors, stories, analogies, etc. The specific criterion is the number of narrative elements employed.<br>1.2 The doctor adopts a patient-centered narrative framework for explanations.<br>Evaluation method: Determine whether the explanation reflects a patient-centered narrative framework. The quantitative criterion is a binary judgment, with 1 indicating presence and 0 indicating absence.<br>2.Empathy<br>2.1 The doctor verbally expresses understanding and support for the patient's emotions.<br>Evaluation method: Count the frequency of empathy-expressing statements made by the doctor, such as "I understand how you feel." The criterion is the frequency of empathy expressions.<br>2.2 The doctor acknowledges the patient's emotional experience and connects it to their symptoms.<br>Evaluation method: Observe whether the doctor asks about or acknowledges the patient's emotional state and whether they connect it to the patient's symptoms or condition. The quantitative criterion is a binary judgment, with 1 indicating presence and 0 indicating absence.<br>3.Personalization<br>3.1 The doctor provides customized medical advice based on the patient's specific circumstances and living environment.<br>Evaluation method: Based on factors such as the patient's lifestyle,

	occupation, and family background, a Likert scale is used for quantitative evaluation (1 point for generalized advice, 5 points for highly personalized advice).<br>3.2 The doctor considers the patient's personal preferences when providing advice.<br>Evaluation method: Determine whether the advice includes customization that takes the patient's preferences into account. The quantitative criterion is a binary judgment, with 1 indicating presence and 0 indicating absence.<br>4.Clarity<br>4.1 The frequency with which the doctor explains medical terminology.<br>Evaluation method: Count the number of times the doctor explains medical terms as a measure of clarity in communication.

2.2 Coding schema design

A coding schema was developed by domain experts in medical communication and linguistics to operationalize four narrative dimensions: storytelling, empathy, personalization, and clarity, following the structure summarized in Supplementary Table S1. Within this framework, storytelling was assessed through (i) narrative frequency and (ii) the presence of a patient-centered narrative framework; empathy through (i) verbal expressions of emotional understanding and support and (ii) emotion–symptom linkage; personalization through (i) an overall personalization score and (ii) consideration of patient preferences; and clarity through terminology explanation frequency. Each indicator was defined using observable linguistic cues to improve consistency and reproducibility.

The schema was designed to minimize subjective interpretation by specifying explicit inclusion criteria and observable linguistic cues for each coding item. The full coding rubric, including definitions and examples, is provided in Supplementary Table S1 and S4.

Table S4. Coding schema for narrative-based communication skills

**Skill**	**Definition**	**Quantifiable Indicator**	**Coding Method**	**Scale**
**storytelling**	Use of narratives, analogies, or metaphors to explain medical concepts.	Narrative elements used (e.g., metaphors, stories, analogies).	Frequency Count (per response)	Count (e.g., 0, 1, 2, etc.)
	Use of a patient-centered narrative framework in explanations.	Presence of patient-centered narrative framework (Yes/ No).	Binary Coding (Yes/ No)	Yes = 1, No = 0
**Empathy**	Verbal expressions of emotional	Frequency of empathetic statements (e.g., “I	Frequency Count (per response)	Count (e.g., 0, 1, 2, etc.)

Skill	Definition	Quantifiable Indicator	Coding Method	Scale
	understanding and support.	understand how you feel”).		
	Acknowledgement of the patient’s emotional experience to symptoms.	Presence of emotion-symptom linkage (Yes/ No).	Binary Coding (Yes/ No)	Yes = 1, No = 0
**Personalization**	Tailoring medical advice to the patient’s specific situation or life context.	Overall personalization score.	Likert-style rating	1-5
	Consideration of the patient’s expressed preferences in advice.	Presence of preference-sensitive guidance (Yes/ No).	Binary Coding (Yes/ No)	Yes = 1, No = 0
**Clarity**	Jargon and medical terminology used during consultations.	Frequency of used jargon terms.	Frequency Count (e.g., number of medical terms).	Count (e.g., 0, 1, 2, etc.)
	Explanation of medical terminology in patient-usable language.	Frequency of terminology explanations.	Frequency Count per response.	Count (e.g., 0, 1, 2, etc.)

To assess the reliability of the AI-assisted annotation pipeline, we conducted an independent double-coding audit during the adjudication process. A random subset of 100 consultations (corresponding to 300 response units across the three response conditions) was independently coded by a domain expert in health communication and medical humanities, who applied the full annotation rubric without access to the initial AI-generated labels and while blinded to response source (doctor, unprompted AI, or prompted AI).

Agreement between provisional AI annotations and independent human coding was evaluated prior to adjudication. Across variables, observed agreement ranged from 0.889 to 1.000, while chance-corrected agreement (Cohen’s κ) ranged from 0.658 to 1.000.

Discrepancies were subsequently resolved through expert adjudication, and all analyses in the main manuscript are based on adjudicated labels. We further quantified the proportion of provisional AI labels modified during adjudication; for example, the modification rate was 3.7% for the presence of a patient-centered narrative framework. Variable-specific modification rates are provided in Supplementary Table S5.

Table S5. Cohen's kappa, observed agreement, and change rate between human and AI coding across variables

Variable	Kappa	Agreement	Change rate
**patient-centered narrative framework**	0.886	0.963	0.037
**emotion-symptom linkage**	1.000	1.000	0.000
**consideration of patient preferences**	0.658	0.889	0.111

2.3 Normalization and analytic controls

To account for variability in response length, all frequency-based coding metrics were normalized by token count. Token length was derived from the segmented text produced during preprocessing and was incorporated either through explicit normalization or as an offset term in regression models.

Reply length varied substantially across response conditions, making token-length adjustment necessary for fair comparison. Supplementary Table S6 therefore reports the distribution of reply length by condition. Clinician replies were shortest on average, the unprompted model was intermediate, and the prompted model was longest. Supplementary Table S7 presents normalized narrative communication density per 1,000 tokens across conditions. As shown, the prompted model consistently outperformed both clinicians and the unprompted model across all three metrics: narrative frequency , verbal empathy, and terminology explanations. These descriptives contextualize the normalization strategy reported in the main analyses and show that the prompted condition's advantage cannot be attributed simply to being the longest response condition.

Table S6. Distribution of reply length (total tokens excluding punctuation) across response conditions

Condition	N	Mean	SD	Median	IQR	Min	Max
**doctor**	1000	89.281	104.5408955	57	83.25	4	1156
**unprompted model**	1000	298.145	135.9168767	278	186.25	47	887
**prompted model**	1000	378.358	132.963646	350.5	158.5	89	973

Table S7. Normalized narrative communication density per 1,000 tokens by condition.

	doctor	unprompted model	prompted model
**Narrative frequency**	0.347218333	0.040248872	9.697165119
**Verbal empathy**	1.030454408	1.455667544	5.25956898
**Terminology explanations**	3.371378009	4.484395177	5.235781984

In addition, domain-term density—defined as the proportion of medical domain terms relative to total tokens—was computed for each response and included as a text-level control variable. This measure served as a proxy for medical complexity and readability, helping to isolate narrative communication effects from variation in technical language use.

3 Statistical Modeling and Inference Framework

3.1 Statistical modeling framework

Comparative analyses were conducted using count-based regression models, with narrative metric counts as dependent variables. Baseline specifications employed

Poisson regression with a log link function and a token-length offset to model rate-based outcomes. Response type (doctor, unprompted AI, prompted AI) was included as a primary explanatory factor.

To assess whether the target event is rare and to establish a descriptive baseline, we used an intercept-only Poisson model with an exposure offset. Let Y_i denote the event count in the i-th consultation and let the observable "amount of opportunity" for that consultation be the total number of tokens after doctor tokenization, $exposure_i$. The model is specified as

$$Y_i \sim Poisson(\mu_i)$$
$$log_{\mu_i} = \beta_0 + log(exposure_i)$$

Here μ_i is the expected event count for consultation i; β_0 is the intercept and represents the average event rate per unit exposure (for example, per token); $log(exposure_i)$ is included as an offset to adjust for differences in consultation length. The model assumes that events occur independently and randomly within each unit of exposure and that counts follow a Poisson distribution. In the intercept-only specification there are no covariates; $exp(\beta_0)$ therefore directly gives the average per-unit rate λ i.e. the probability of the event per token. The model-predicted expected count is

$$\hat{\mu}_i = exposure_i \times \hat{\lambda}$$

For interpretability, results are also reported as the expected number of events per 100 tokens, which expresses the model-estimated standardized event density. In this study the model is used solely as a descriptive baseline for rarity: by comparing the Poisson model's expectations (e.g. the theoretical zero proportion implied by the estimated mean) with observed data (e.g. empirical zero proportion and sample mean), we can evaluate whether the event behaves like a Poisson rare event.

Where appropriate, alternative model specifications were explored to assess robustness, including negative binomial formulations to address overdispersion. Model fitting was performed consistently across all narrative dimensions to ensure comparability of estimates.

3.2 Uncertainty estimation and confidence intervals

Statistical uncertainty was quantified using nonparametric bootstrap resampling. Specifically, we employed the percentile bootstrap method: treating the observed sample as an approximation of the population, we performed repeated sampling with replacement (B = 2000 bootstrap replicates, with a fixed random seed for reproducibility). For each resample, we computed the statistic of interest—such as the sample mean or a binary proportion—and took the 2.5th and 97.5th percentiles of the

resulting empirical distribution as the 95% bootstrap confidence interval. This approach does not rely on normality or any specific parametric distribution and yields robust interval estimates for means and proportions in large samples. In the present work, we report bootstrap 95% CIs alongside observed means (and zero-event proportions) to provide a direct reflection of sampling uncertainty.

For proportion-based and binary outcomes, Wilson confidence intervals were computed to provide stable interval estimates under conditions of low prevalence or skewed distributions. The Wilson confidence interval is an analytic method for estimating binomial proportions that aims to provide a more reliable interval than the simple Wald interval.Its derivation is linked to the score test (Wilson score): instead of directly applying a normal approximation around $\hat{p}$, the method re-centers and rescales the proportion using a more accurate approximation to the binomial distribution, which makes the interval more stable when the sample size is small or the observed proportion is near 0 or 1.

In closed form (for a two-sided $1-\alpha$ interval with $z_{1-\alpha/2}$,typically $z \approx 1.96$ for 95% CI), let $p = \hat{p} = \frac{k}{n}$ .Define

$$denom = 1 + \frac{z^2}{n}$$

$$center = \frac{p + \frac{z^2}{2n}}{denom}$$

$$half = \frac{z\sqrt{\frac{p(1-p)}{n} + \frac{z^2}{4n^2}}}{denom}$$

The Wilson interval is then

$$[center - half, center + half]$$

These approaches were selected to improve reliability relative to asymptotic normal approximations.

3.3 Detailed Quantitative Analysis of Narrative Communication

Table S8. Descriptive statistics for human doctors

	**Human doctors**			
	**observed mean**	**bootstrap**	**zero proportion**	**Wilson （zero proportion）**
**Narrative frequency**	0.031	[0.019, 0.044]	0.974	[0.962, 0.982]
**Verbal empathy**	0.092	[0.070, 0.116]	0.932	[0.9147, 0.9460]
**Terminology explanations**	0.301	[0.259, 0.348]	0.811	[0.786, 0.834]
	**observed mean**	**bootstrap**	**zero proportion**	**Wilson （observed mean）**
**Emotion–symptom linkage**	0.026	[0.017, 0.036]	0.074	[0.0178, 0.0378]
**Consideration of patient preferences**	0.15	[0.128, 0.172]	0.85	[0.129, 0.173]
**Patient-centered narrative framework**	0.499	[0.469, 0.530]	0.501	[0.468, 0.530]

	Human doctors			
	observed mean	bootstrap	zero proportion	Wilson (zero proportion)
	observed mean	bootstrap	standard deviation	
**Overall personalization score**	1.674	[1.612, 1.738]	0.615	

Table S9. Descriptive statistics for unprompted AI

	Unprompted AI			
	observed mean	bootstrap	zero proportion	Wilson (zero proportion)
**Narrative frequency**	0.012	[0.006,0.019]	0.988	[0.979,0.993]
**Verbal empathy**	0.434	[0.393, 0.477]	0.688	[0.659, 0.716]
**Terminology explanations**	1.337	[1.256, 1.421]	0.383	[0.353, 0.414]
	observed mean	bootstrap	zero proportion	Wilson (observed mean)
**Emotion–symptom linkage**	0.144	[0.124, 0.165]	0.856	[0.1236, 0.1671]
**Consideration of patient preferences**	0.206	[0.181, 0.231]	0.794	[0.1821, 0.2322]
**Patient-centered narrative framework**	1	[1.000, 1.000]	0	[0.996, 1.000]
	observed mean	bootstrap	standard deviation	
**Overall personalization score**	2.846	[2.767, 2.922]	0.42	

Table S10. Descriptive statistics for prompted AI

	Prompted AI			
	observed mean	bootstrap	zero proportion	Wilson (zero proportion)
**Narrative frequency**	3.669	[3.576,3.762]	0	[0, 0.00383]
**Verbal empathy**	1.99	[1.953, 2.027]	0	[0, 0.00383]
**Terminology explanations**	1.981	[1.901, 2.061]	0.153	[0.132, 0.177]
	observed mean	bootstrap	zero proportion	Wilson (observed mean)
**Emotion–symptom linkage**	0.991	[0.985, 0.996]	0.009	[0.98298, 0.99526]
**Consideration of patient preferences**	0.542	[0.512, 0.573]	0.458	[0.511, 0.573]
**Patient-centered narrative framework**	1	[1.000, 1.000]	0	[0.996, 1.000]
	observed mean	bootstrap	standard deviation	
**Overall personalization score**	3.833	[3.775, 3.895]	0.25	

3.3.1 Storytelling

Narrative frequency: For rare behaviors such as spontaneous use of analogies or metaphors, human doctors exhibited extremely low counts, with a mean of 0.031 narrative instances per dialogue (95% bootstrap CI = [0.019, 0.044]). Correspondingly, 97.4% of dialogues contained zero occurrences (Wilson 95% CI = [0.962, 0.982]). Under a Poisson model parameterized by the observed mean, the expected zero proportion is 96.9%, which closely aligns with the empirical value, indicating that the rarity of this behavior is well captured by a Poisson rare-event process. Although the deviance-to-degrees-of-freedom ratio(0.214) was below one, suggesting mild underdispersion, this reflects lower-than-expected variability rather than model misspecification, and supports the interpretation of narrative use as a consistently rare behavior among human doctors.

Unprompted AI exhibited an even lower frequency of narrative usage, with only 12 occurrences across 1,000 dialogues (mean = 0.012; bootstrap 95% CI = [0.006,

0.019]). The observed zero proportion was 98.8% (Wilson 95% CI = [0.979, 0.993]), which almost perfectly matched the Poisson-expected zero proportion of 98.8% derived from the sample mean. The variance-to-mean ratio was close to unity, indicating near-ideal conformity to Poisson assumptions and reinforcing the conclusion that narrative expression was virtually absent in unprompted AI responses.

In contrast, prompted AI showed a marked departure from a rare-event regime, producing 3,669 narrative instances across 1,000 dialogues (mean = 3.669; bootstrap 95% CI = [3.576, 3.762]). Under a Poisson model with this mean, the expected zero proportion would be approximately 2.6%; however, no zero-event dialogues were observed, with the upper bound of the Wilson confidence interval remaining below 0.4%. Additionally, the variance-to-mean ratio (≈ 0.62) indicated pronounced underdispersion, reflecting highly consistent narrative generation across dialogues. Together, these patterns demonstrate a clear transition from Poisson-governed rare events to a stable, high-frequency communicative behavior following targeted training.

Patient-centered narrative framework: Human doctors used patient-centered narrative framework in 49.9% of dialogues (499 out of 1,000 dialogues; mean proportion = 0.499), with a Wilson 95% confidence interval of [0.468, 0.530] and a nonparametric bootstrap 95% CI of [0.469, 0.530]. In this study, this variable refers specifically to whether the explanation reflects a patient-centered narrative framework, as defined in Supplementary Table S1, rather than to the full construct of patient-centeredness. The close alignment between these intervals and the relatively narrow range (≈ ±3%) indicate stable and reliable estimation. This suggests that patient-centered narrative strategies are a prevalent and consistent aspect of human doctors' communication, reflecting their tendency to explain diseases and treatments from the patient's perspective.

Unprompted AI already demonstrated 100% patient-centered narrative framework (1,000/1,000 dialogues), with a Wilson 95% CI of [0.996, 1.000] and a bootstrap 95% CI of [1.000, 1.000], showing virtually no uncertainty and extremely high stability. After targeted training, the AI maintained a perfect 100% patient-centered propensity (prop = 1.000), with fully consistent confidence intervals, indicating that training preserved its linguistic consistency while enhancing its structured capacity to adopt patient perspectives. Compared to human doctors, the AI demonstrates not only a higher proportion of patient-centered responses but also superior reproducibility and standardization. Post-training, the AI progresses from merely adhering to patient-oriented language norms to actively embodying humanistic narrative competence, naturally integrating patient concerns, emotional empathy, and explanatory clarity. Overall, training enhanced the AI's narrative capability without compromising patient orientation, significantly surpassing human doctors in both consistency and narrative quality.

3.3.2 Empathy

Verbal empathy: Human doctors exhibited low frequency of explicit verbal empathy, with a mean of 0.092 occurrences per dialogue (92 instances across 1,000 dialogues), and a nonparametric bootstrap 95% confidence interval of [0.070, 0.116]. Most dialogues (93.2%) contained zero explicit empathetic expressions (Wilson 95% CI = [0.915, 0.946]), slightly higher than the Poisson expectation based on the mean (≈91.2%), indicating mild clustering. The variance-to-mean ratio (≈1.562) suggests mild over-dispersion, reflecting that while verbal empathy is generally rare, it tends to cluster in a small subset of dialogues rather than being purely random. Overall, human doctors demonstrate empathy sporadically, with most dialogues containing no explicit empathetic statements.

Unprompted AI displayed higher but uneven empathy, with a mean of 0.434 per dialogue (434 instances across 1,000 dialogues) and a bootstrap 95% CI of [0.393, 0.477]. The zero proportion remained high at 68.8% (Wilson CI ≈ [0.659, 0.716]), and the variance-to-mean ratio was approximately 1.20, indicating moderate over-dispersion. This pattern reflects clustered deployment: empathy occurs frequently in some dialogues but is absent in most, demonstrating sporadic yet occasionally strong empathetic expression.

After training, AI produced consistent and common empathy, with a mean of 1.99 per dialogue (1,990 instances across 1,000 dialogues) and a bootstrap 95% CI of [1.953, 2.027]. The zero proportion dropped to 0, and the variance-to-mean ratio decreased to 0.176, indicating under-dispersion and highly uniform expression across dialogues. Compared to the theoretical Poisson expectation (≈13.7% zeroes), the absence of zero-count dialogues highlights a substantial shift from a rare-event distribution to a stable, common behavior. This demonstrates that training substantially improved both the frequency and consistency of AI-generated empathy, transforming it from sporadic to reliably present across all dialogues.

Emotion–symptom linkage: Human doctors rarely connected patients' emotional experiences to symptoms, with only 26 out of 1,000 dialogues exhibiting this behavior (2.6%; Wilson 95% CI = [0.0178, 0.0378], bootstrap 95% CI = [0.017, 0.036]). The consistency between these confidence intervals indicates stable estimation despite the low number of positive cases. Overall, approximately 97.4% of human doctor dialogues did not explicitly link emotions to symptoms, suggesting that clinicians seldom make such connections during routine clinical communication, potentially influenced by consultation context, Q&A format, or annotation standards.

Unprompted AI performed this linkage more frequently, appearing in 144 of 1,000 dialogues (14.4%; Wilson 95% CI = [0.1236, 0.1671], bootstrap 95% CI = [0.124, 0.165]). The narrow and consistent confidence intervals indicate reliable estimation. Although this proportion is substantially higher than the human baseline, the behavior remains sporadic, with the majority of dialogues (≈85.6%) still lacking

explicit emotion-symptom connections. This pattern suggests that the unprompted AI can generate emotion-symptom linkages in certain contexts but does not consistently do so across all dialogues.

After training, AI nearly saturated this behavior, with 991 out of 1,000 dialogues positive (99.1%; Wilson 95% CI = [0.983, 0.995], bootstrap 95% CI = [0.985, 0.996]). The extremely narrow confidence intervals reflect exceptional stability and reproducibility. Compared to human doctors (≈2.6%) and unprompted AI (≈14.4%), the prompted AI demonstrates a dramatic improvement in explicitly linking patient emotional experiences to symptoms, indicating that training markedly enhanced its capability to integrate emotion-symptom connections across dialogues. Overall, post-training AI far surpasses both the human baseline and its pre-prompted state in this specific aspect of empathetic communication.

3.3.3 Personalization

Overall personalization score: Human doctors achieved a mean personalization score of 1.674 on a 1–5 scale (bootstrap 95% CI = [1.612, 1.738]; SD = 1.029), reflecting generally generic recommendations. The standard deviation relative to the mean (≈61.5%) indicates moderate heterogeneity, suggesting that while most recommendations were generic, a subset of doctors provided more personalized advice. The narrow bootstrap confidence interval demonstrates that the mean estimate is stable and reproducible.

Unprompted AI scored 2.846 (95% CI = [2.767, 2.922]; SD = 1.202), falling in the upper-middle range of the 1–5 scale. Compared to human doctors, this represents a substantial improvement, with the bootstrap CI not overlapping the human mean. The ratio of standard deviation to mean (≈0.42) indicates moderate variability across dialogues, with some recommendations being highly personalized while others remain relatively generic. Overall, the unprompted AI demonstrates a clear advantage in tailoring advice to patient-specific conditions.

Prompted AI achieved a mean score of 3.833 (95% CI = [3.775, 3.895]; SD = 0.959), substantially above both human doctors (≈1.67) and unprompted AI (≈2.85). The relatively small standard deviation (≈25% of the mean) combined with the narrow confidence interval indicates that this high level of personalization is consistently maintained across dialogues. Post-training, the AI demonstrates reliably high personalization, with significantly improved ability to tailor recommendations to individual patient conditions

Consideration of patient preferences: Human doctors explicitly considered patient preferences in only 150 out of 1,000 dialogues (15.0%; Wilson 95% CI = [0.129, 0.173], bootstrap 95% CI = [0.128, 0.172]). The narrow and consistent confidence intervals indicate stable estimation. A frequency of 15% reflects a relatively low-occurrence behavior: in routine consultations, doctors rarely

proactively inquire about or adjust recommendations according to patient preferences.

Unprompted AI performed slightly better, explicitly referencing patient preferences in 206 of 1,000 dialogues (20.6%; Wilson 95% CI = [0.182, 0.232], bootstrap 95% CI = [0.181, 0.231]). The narrow confidence intervals indicate reliable estimation. Compared to the human baseline (≈15%), the unprompted AI already demonstrates a higher tendency to accommodate patient preferences, although the majority of dialogues still lack explicit preference consideration.

Post-training AI substantially improved, showing preference consideration in 542 out of 1,000 dialogues (54.2%; Wilson 95% CI = [0.511, 0.573], bootstrap 95% CI = [0.512, 0.573]). The narrow and consistent confidence intervals indicate that this high occurrence rate is estimated robustly. Compared to human doctors (≈15%) and unprompted AI (≈20.6%), the prompted AI demonstrates a significant enhancement in sensitivity and execution of preference-informed recommendations, explicitly accounting for patient preferences in over half of all dialogues.

3.3.4 Clarity

Terminology explanations: Human doctors provided terminology explanations infrequently, with a total of 301 occurrences across 1,000 dialogues (mean = 0.301 per dialogue; bootstrap 95% CI = [0.259, 0.348]). The distribution is highly skewed: the median and interquartile range are 0, and 81.1% of dialogues contained no terminology explanations (zero proportion = 0.811, Wilson 95% CI ≈ [0.786, 0.834]). Among dialogues containing explanations, the mean number of explanations per dialogue was 1.59, indicating that when explanations occur, they are typically 1–2 per dialogue. The variance-to-mean ratio is 1.75 (>1), indicating over-dispersion, and the observed zero proportion is slightly higher than the Poisson expectation (exp(−0.301) ≈ 0.740), reflecting mild zero-inflation. Overall, terminology explanations by human doctors are generally low-frequency but not entirely rare, with heterogeneous distribution across dialogues.

Unprompted AI improved coverage substantially, with 1,337 terminology explanations across 1,000 dialogues (mean = 1.337; bootstrap 95% CI = [1.256, 1.421]). The median is 1 (interquartile range = 2), and 61.7% of dialogues contained at least one explanation (zero proportion ≈ 38.3%, Wilson 95% CI ≈ [0.353, 0.414]). The mean number of explanations in non-zero dialogues is 2.17, showing that dialogues with explanations often contain multiple instances. The variance-to-mean ratio is 1.35 (>1), indicating over-dispersion, and the observed zero proportion is higher than the Poisson expectation (exp(−1.337) ≈ 0.263), reflecting some zero-inflation. These patterns indicate that the unprompted AI is more active in providing terminology explanations than human doctors and delivers more comprehensive explanations when they occur, but output remains heterogeneous across dialogues.

Post-training AI further increased both frequency and consistency, producing 1,981

terminology explanations across 1,000 dialogues (mean = 1.981; bootstrap 95% CI = [1.901, 2.061]). The median is 2 (interquartile range = 2), with approximately 84.7% of dialogues containing at least one explanation (zero proportion ≈ 15.3%, Wilson 95% CI ≈ [0.132, 0.177]). The mean number of explanations in non-zero dialogues is 2.34, close to the overall mean, indicating balanced and stable explanatory behavior. The variance-to-mean ratio is 0.85 (<1), suggesting slight under-dispersion and minimal fluctuation across dialogues. The observed zero proportion is close to the Poisson expectation (exp(−1.981) ≈ 0.138), indicating minimal zero-inflation. Overall, the prompted AI demonstrates proactive, frequent, and consistent terminology explanation behavior, reflecting improved understanding of patient needs and enhanced clarity in communication.

3.4 Model diagnostics and robustness checks

Figure S1. Per-Year Observed vs Poisson-Expected Zero Proportions — Clarity (Terminology explanations)

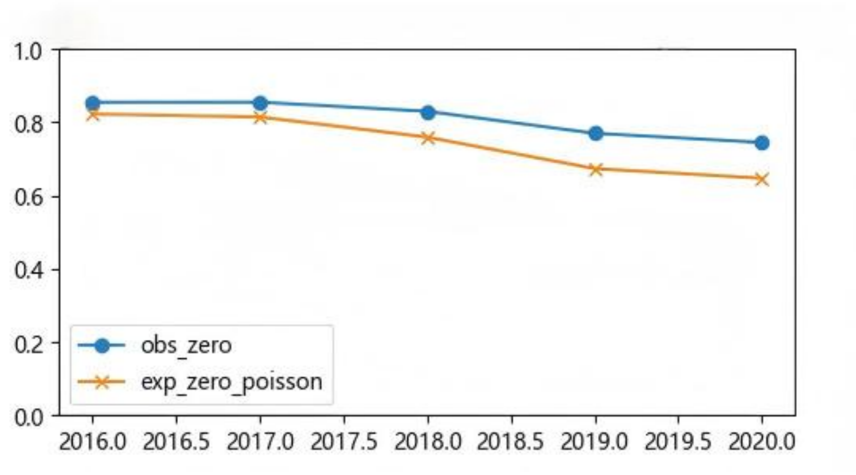

Figure S2. Observed vs Poisson-Expected Zero Proportions — Clarity (Terminology explanations)

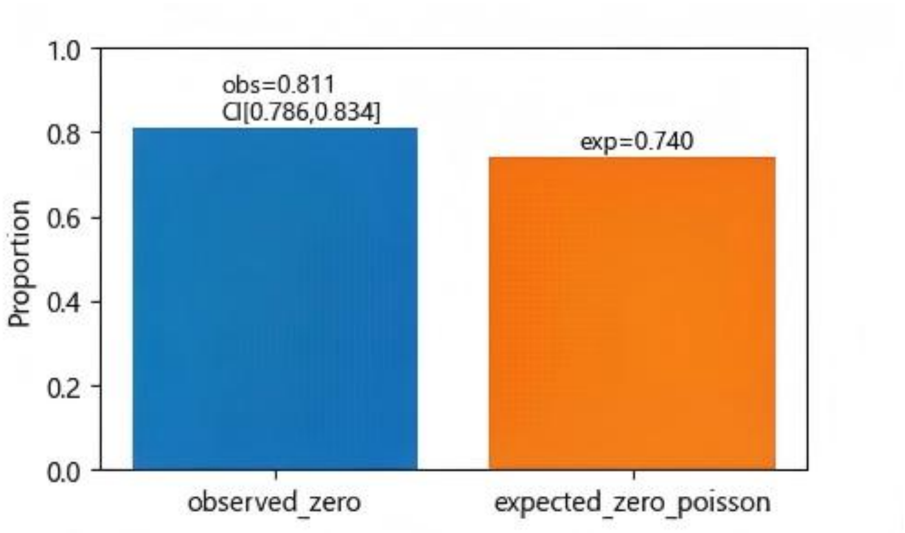

Figure S3. Bootstrap Distribution of Mean Counts — Clarity (Terminology explanations)

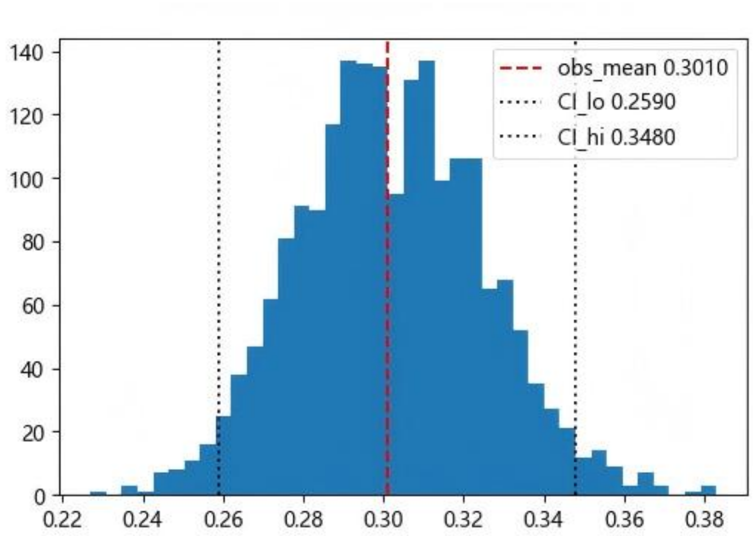

Model diagnostics were conducted to evaluate dispersion properties and the prevalence of zero counts across outcome variables. The ratio of variance to mean and the proportion of zero observations were examined to assess the adequacy of Poisson assumptions.

Sensitivity analyses were performed by varying model specifications and exclusion criteria to confirm the stability of estimated effects. Across these checks, qualitative patterns and comparative relationships remained consistent, supporting the robustness of the reported findings.
